# An Integrated Pixel-Level Reflectance Adjustment (IPRA) for Harmonizing GF-1/6 WFV and Sentinel-2 MSI Data

**DOI:** 10.3390/s26061759

**Published:** 2026-03-10

**Authors:** Jianli Shi, Xingfa Gu, Yan Liu, Yaozong Ding, Qian Zhang, Yang Yang

**Affiliations:** 1 Aerospace Information Research Institute, Chinese Academy of Sciences, Beijing 100094, China; shijianli23@mails.ucas.ac.cn (J.S.); dingyaozong22@mails.ucas.ac.cn (Y.D.); zhangqian221@mails.ucas.ac.cn (Q.Z.); yangyang231@mails.ucas.ac.cn (Y.Y.); 2 University of Chinese Academy of Sciences, Beijing 100049, China; 3 School of Geography and Remote Sensing, Guangzhou University, Guangzhou 510006, China

**Keywords:** GF-1/6 WFV, Sentinel-2 MSI, harmonizing, pixel-level

## Abstract

**Highlights:**

**What are the main findings?**

- An integrated pixel-level reflectance adjustment (IPRA) method for GF-1/6 WFV is developed. The proposed method outperforms traditional normalization approaches, with improved temporal stability in cross-sensor radiometric normalization.
- A spatial heterogeneity detection mechanism is proposed to effectively mitigate geometric distortions and enable robust radiometric normalization.
- A time-weighted fitting model using Weighted Least Squares (WLS) is established to effectively mitigate uncertainty associated with temporal sensor degradation and ensure long-term radiometric consistency.

**What are the implications of the main findings?**

- The proposed method effectively mitigates geometric distortions and temporal decay inherent in GF-1/6 WFV sensors and thereby provides a robust pixel-level solution for cross-sensor radiometric normalization.
- By using the IPRA method for radiometric normalization, an integrated multi-source dataset with higher temporal frequency can be created, and it supports more effective long-term monitoring of surface dynamics.

**Abstract:**

This study proposes an integrated pixel-level reflectance adjustment (IPRA) method using Sentinel-2 MSI as the reference to address radiometric discrepancies in GF-1/6 WFV imagery, particularly caused by sensor decay and geometric distortions. The proposed IPRA method leverages time-series data and a spatial heterogeneity detection mechanism to effectively mitigate geometric distortions. Furthermore, it incorporates a weighted linear regression (WLR) model to weight pixels based on their temporal decay characteristics. The results demonstrate that IPRA outperforms existing methods (i.e., IRMAD, HM, and TRA) in radiometric consistency, yielding smaller radiometric discrepancies relative to Sentinel-2 MSI. Specifically, NAE decreased by 42.9% (from 0.319 to 0.182), RMSE decreased by 37.3% (from 0.051 to 0.032), PSNR improved from 25.906 dB to 30.195 dB, and the SC value approached the ideal value of 1 (from 1.540 to 1.001). In conclusion, the IPRA method provides a robust solution for normalizing GF-1/6 WFV imagery and thus facilitates its cross-sensor applications.

## 1. Introduction

Fine spatial resolution satellite sensors—such as Sentinel-2 Multispectral Imager (MSI), Landsat-8/9 Operational Land Imager (OLI), SPOT-5 High Resolution Geometric (HRG), and GF-1/6 Wide Field of View (WFV)—provide remote sensing imagery with global coverage, high temporal resolution, and multispectral imaging capabilities. These datasets are freely accessible and play a crucial role in monitoring the Earth system and global change [1], including land-cover change detection [2], agricultural monitoring [3], forest ecosystem management [4], water resource assessment [5], urban ecology [6], and disaster response [7]. GF-1 and GF-6, together with Sentinel-2, feature relatively high revisit frequencies, and their synergistic use can effectively increase observation frequency.

In recent years, many scholars have employed synergistic approaches using satellite data for various applications. For example, Kim et al. [8] jointly employed Sentinel-2 and Landsat-8 to monitor large-scale forest fires in Korea and thereby effectively obtained accurate ground information over extensive areas. Fang et al. [9] integrated Sentinel-2 and Landsat-8 to predict forest variables, thereby improving cloud-free observation frequency while leveraging the spectral advantages of different sensors. Similarly, Nishan et al. [10] addressed the challenges of long revisit cycles and frequent cloud contamination in Landsat data by combining Landsat with Sentinel-2 for crop water modeling. Liu et al. [11] constructed a long-term time series by combining GF-1 and Sentinel-2 data for apple orchard yield estimation. Zhang et al. [12] employed a joint dataset of GF-series and Sentinel-2 satellites for fire monitoring and achieved higher accuracy. These studies demonstrate that cross-sensor integration represents a key trend for improving monitoring accuracy and supporting long-term Earth observation applications. While the synergistic application of multi-source remote sensing data offers significant advantages, radiometric discrepancies remain the most critical obstacle, making radiometric normalization an essential process in these applications.

GF-1, launched in April 2013 as the first CHEOS (China High-resolution Earth Observation System) satellite, is equipped with four Wide Field of View (WFV) cameras, covering four spectral bands from the visible to the near-infrared range [13,14]. GF-6, launched in June 2018, carries a 16 m multispectral WFV camera and offers both high spatial resolution and large coverage [15,16]. GF-1/6 satellites achieve a four-day revisit cycle in side-looking mode. GF-1 and GF-6 have similar sensor configurations, which facilitate multi-satellite synergy for wide-area Earth observation [15]. Due to their wide-area observation capabilities with high spatial resolution, GF-1/6 WFV data have been widely applied in water monitoring [17], crop classification [18], and forest management [19]. Both GF-1/6 WFV sensors lack onboard calibration and rely on annual vicarious calibration from the China Center for Resources Satellite Data and Application (CRESDA) [20,21]. With GF-1 exceeding its design life [15] and GF-6 nearing its end [21], sensor aging further degrades radiometric quality and causes substantial bias. Li et al. [22] assessed the long-term radiometric quality of GF-1/6 satellites and found that both GF-1 and GF-6 WFV exhibit inconsistent accuracy, with varying differences across spectral bands. These findings indicate complex radiometric discrepancies in the WFV sensors. Liu et al. [23] and Wang et al. [24] performed calibrations on GF-1/6 WFV by considering only spectral response factors. Their results revealed that radiometric discrepancies exist and tend to decay over time. Therefore, to obtain accurate surface reflectance data and enable cross-sensor integration, it is necessary to enhance the radiometric accuracy of these sensors, for which relative radiometric normalization provides an effective solution.

The Sentinel-2 mission (2A launched in 2015, 2B in 2017) carries a 13-band MSI sensor, including red, green, blue, and NIR bands, with a 10 m spatial resolution and a 5-day revisit cycle, which is comparable to GF-1/6 WFV [25,26]. Sentinel-2 products have been rigorously calibrated and validated, and have achieved high radiometric accuracy (absolute uncertainty < 5%) and long-term radiometric stability (<1%/year degradation), as well as sub-pixel geometric registration accuracy (~0.3 pixel) [27,28]. It provides free global access under the Copernicus program [29]. Given these advantages, Sentinel-2 imagery has been widely used as a reference for radiometric normalization of other satellite datasets [30,31].Considering Sentinel-2’s superior radiometric stability, geometric quality, and openness, it is an ideal reference for GF-1/6 WFV sensors.

Relative radiometric normalization (RRN) is a remote sensing image correction approach that addresses sensor radiometric discrepancies caused by non-surface factors by using a highly accurate reference sensor [32,33]. RRN methods can be broadly classified into sparse RRN (SRRN) and dense RRN (DRRN) approaches [34,35].SRRN identifies pseudo-invariant features (PIFs) or stable ground targets to establish pixel-to-pixel correspondences between the reference and target images, by using regression models for correction [36]. Common approaches include Simple Regression (SR) [37], PIF-based regression [38], Automatic Scatterplot Controlled Regression (ASCR) [39], and Iteratively Reweighted Multivariate Alteration Detection (IRMAD) [40]. DRRN adjusts the target image’s statistical distribution to match the reference by aligning brightness and contrast without requiring pixel-level registration [41]. Common approaches include Minimum–Maximum normalization (MM) [41], Mean–Standard Deviation normalization (MS) [32], and Histogram Matching (HM) [42]. While SRRN needs high-accuracy registration for optimal performance, DRRN is more tolerant of geometric misalignment but may lose fine details [43]. Shang et al. [44] developed a time-series-based reflectance adjustment (TRA) approach tailored for harmonizing Landsat 8 and Sentinel-2 data. However, this method fails to account for the inherent limitations specific to the GF satellite series.

To date, many studies have been dedicated to the radiometric normalization of GF-1/6 WFV, and they primarily employ the previously described methods. Huang et al. [45] proposed a Control No-Change Set (CNCS) method based on IRMAD for radiometric normalization of GF-1 WFV imagery, which optimized the selection of pseudo-invariant features. Yang et al. [46] applied kernel canonical correlation analysis (kCCA) for radiometric normalization of GF-1 imagery, which took potential nonlinear spectral differences between sensors into account. Huang et al. [47] employed a regularized IRMAD method for radiometric normalization of GF-1 WFV imagery, aiming to improve the selection of PIFs. Xu et al. [48] proposed a change vector–based index for the extraction of PIFs. These methods belong to SRRN. Their core lies in the selection and optimization of PIFs using various algorithms. As pixel-based approaches, they extract PIFs by calculating specific metrics between corresponding pixels and rely heavily on point-to-point correlation. At the pixel scale, a displacement of even a single pixel can lead to abrupt changes in reflectance. Consequently, the extraction process often yields false positives when satisfying these metrics; the identified PIFs are statistically compliant but spatially mismatched, leading to the misidentification or omission of true invariant features. Due to the wide swath, large field of view, and lack of Ground Control Fields (GCF), GF-1/6 WFV imagery suffers from geometric distortions, especially at the image edges, and results in positional errors [49,50]. While these approaches achieve some degree of radiometric correction, significant errors still exist in the quantitative results. In contrast, DRRN has seen limited application in GF-1/6 WFV normalization. Taking HM as the representative approach, this method does not require strict geometric registration. It operates by utilizing a non-linear remapping of the Cumulative Distribution Function (CDF) to align the reflectance range of the target image with that of the reference. Nevertheless, in areas characterized by severe geometric distortions, this approach still yields erroneous mapping relationships. Furthermore, due to the quantization discontinuity of reflectance values, such non-linear mapping often triggers histogram breakage, leading to a loss of image detail. Overall, while SRRN is highly sensitive to geometric errors, DRRN offers some tolerance but provides limited correction precision. Furthermore, both of these methods typically operate on a single image pair without taking into account the long-term radiometric decay characteristics of the sensor. TRA method was originally designed for radiometrically stable sensors, such as Landsat and Sentinel-2, to perform long-term time-series adjustments. This method typically assigns equal weights to images from different dates when building fitting models. However, when applied to GF-1/6 WFV data, this equal-weighting strategy fails to account for the significant and unstable decay characteristic of GF sensors and results in undesirable results in long-term reflectance adjustments. Furthermore, TRA method relies on strict geometric registration to extract consistent pixel-level time series. Due to the geometric distortions in GF-1/6 WFV data, the pixel-matching quality is inevitably reduced, which in turn leads to a decline in the accuracy of the radiometric normalization. Therefore, the inherent limitations of GF-1/6 WFV images should be explicitly considered when performing radiometric normalization.

Considering the persistent discrepancies in current radiometric normalization methods, particularly for the inherent geometric distortions and temporal decay of GF-1/6 WFV sensors, this study proposes the IPRA framework. This approach integrates a spatial heterogeneity detection mechanism to enhance the geometric fidelity of normalization results and effectively shields the process from widespread registration errors. It employs a Gaussian-weighted linear regression to address the long-term radiometric decay inherent in GF-1/6 WFV sensors. By integrating these mechanisms, IPRA uniquely improves spatial robustness and radiometric accuracy. To validate its performance, the proposed method is compared against three normalization paradigms: IRMAD, HM and TRA. Results from multiple statistical evaluations show that the proposed IPRA approach achieves higher normalization accuracy and superior image quality. Furthermore, it effectively mitigates the inherent limitations of GF-1/6 WFV data, particularly addressing sensor-specific temporal decay and geometric distortions.

## 2. Study Areas and Data Sources

### 2.1. Study Areas

**Figure 1:**
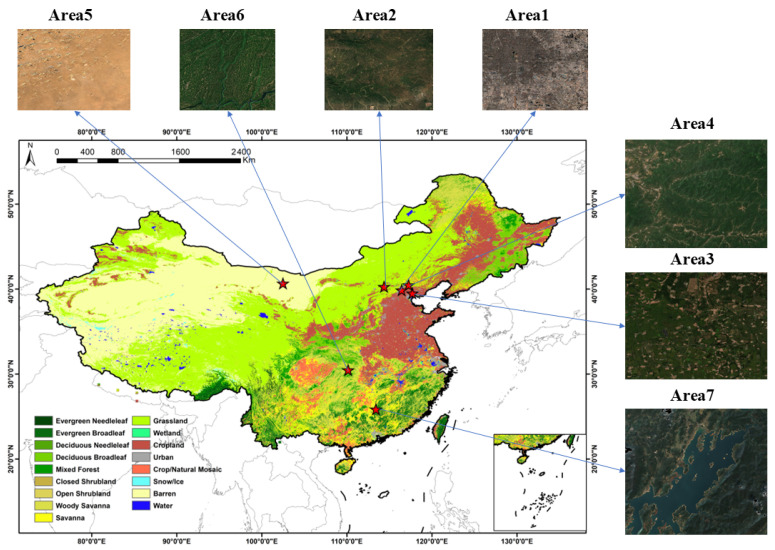
Spatial Distribution of the Study areas.

Because different land cover types exhibit varying radiometric characteristics and biases, representative areas were selected (Figure 1). Seven study areas were selected (Table 1), distributed across China and spanning 100–120° E and 20–45° N. These areas possess diverse land cover types, including forests, grassland, cropland, water bodies, and bareland —as classified by the MODIS International Geosphere-Biosphere Programme (IGBP) product. These areas are characterized by rich land cover diversity and spatial heterogeneity [51,52,53,54]. In these areas, GF-1, GF-6, and Sentinel-2 provide high observation frequency, which allows the acquisition of multi-temporal remote sensing data at each site to support this study.

In each study area, we randomly selected 100 windows and calculated their means and standard deviations for variance analysis. The results of both the mean and variance analyses indicate that the seven study areas exhibit significant radiometric differences (*p* < 0.01), which validates their high representativeness and ability to reflect complex surface reflectance characteristics effectively.

### 2.2. Satellite Surface Reflectance Data

This study uses surface reflectance data from GF-1 WFV, GF-6 WFV, and Sentinel-2 MSI imagery. The selected spectral bands include the visible bands (blue, green, red) and the near-infrared band. GF-1 WFV and GF-6 WFV data are available from the CRESDA website (https://data.cresda.cn). These data are provided as digital numbers (DN), accompanied by calibration coefficients and rational polynomial coefficients (RPC) for orthorectification. Orthorectification was performed using a 30 m DEM as a reference. Subsequently, atmospheric correction was conducted with the Second Simulation of the Satellite Signal in the Solar Spectrum (6S) radiative transfer model to yield surface reflectance data at a spatial resolution of 16 m [55,56,57]. Sentinel-2 MSI imagery was obtained from the COPERNICUS/S2_SR_HARMONIZED dataset processed on the Google Earth Engine (GEE) cloud computing platform. This dataset contains atmospherically corrected surface reflectance data with a spatial resolution of 10 m [58,59]. Cloud-free pixels were identified using the quality assessment (QA) band. In this study, Sentinel-2 bands B2 (blue), B3 (green), B4 (red), and B8 (near-infrared)—corresponding to the blue, green, red, and near-infrared bands of GF-1/6 WFV imagery—were selected for analysis.

## 3. Methodology

### 3.1. Data Preprocessing

The selection of areas was based on land cover types and observation frequency. Considering the slight spectral and radiometric differences between GF-1 WFV and GF-6 WFV [60,61], as well as the limited data available for simultaneous observations between GF-1/6 WFV and Sentinel-2 MSI, we performed normalization separately for GF-1 WFV and GF-6 WFV, with normalization conducted for different years. In areas 1–4, year-round GF-1 WFV and Sentinel-2 MSI imagery from 2020 were selected, while areas 5–7 used GF-6 WFV and Sentinel-2 MSI imagery from 2022.

This study used surface reflectance instead of top-of-atmosphere (TOA) reflectance (Table 2). Compared with TOA reflectance, surface reflectance more accurately represents true land surface information while being less affected by atmospheric conditions, and thus minimizes the impact of temporal differences between images—a factor critical for achieving cross-sensor consistency and comparability. As a result, surface reflectance provided a more stable basis for vegetation index calculation and forest parameter estimation. Use of surface reflectance helped mitigate external environmental effects, normalize inter-sensor differences, and ensure reliable surface monitoring. The calculation procedure for surface reflectance was as follows:

(1) GF-1/6 WFV

GF-1/6 WFV Level-1 data downloaded from CRESDA include solar/satellite angles and RPC information for orthorectification, but users are required to perform geometric, radiometric, and spectral correction [62,63,64]. The blue, green, red, and NIR bands were selected for radiometric normalization. For the GF-1/6 WFV data, orthorectification was conducted based on the RPC model, with the DEM as spatial and topographic reference.

Radiometric calibration converted DN to radiance [62], followed by atmospheric correction using the 6S model to produce surface reflectance products [55].

(1) $$\rho_{TOA}\left(\theta_{s},\theta_{i},\phi \right)=\rho_{a}\left(\theta_{s},\theta_{i},\phi \right)+\frac{\rho_{SR}}{1-\rho_{SR}\cdot S}T\left(\theta_{s}\right)T\left(\theta_{i}\right)$$

In Equation (1), which represents the radiative transfer equation, the 6S model was used for the calculations. $\theta_{s},\theta_{i}$ and ϕ represent the solar zenith angle, observation zenith angle, and the relative azimuth angle between the sun and the sensor, respectively. $\rho_{a}$ refers to the path radiance caused by Rayleigh scattering and aerosol scattering, $\rho_{SR}$ is the surface reflectance, S is the atmospheric hemispherical reflectance, and $T(\theta_{s})$ is the total atmospheric transmittance from the sun to the surface, and $T(\theta_{i})$ is the total atmospheric transmittance from the surface to the satellite.

To achieve spectral adjustment, hyperspectral data are convolved with the spectral response functions (SRF) of target sensors to simulate the band-integrated reflectance, which is then used to establish the conversion relationships, as shown in Equation (2) [65,66]. Spectral conversion is then performed to adjust the GF-1/6 WFV spectrum to match that of Sentinel-2 MSI.

(2) $$\;\;\;\;\bar{\rho_{\lambda }}=\frac{\int \rho_{\lambda }\times {RSR}_{\lambda }d\lambda }{\int {RSR}_{\lambda }d\lambda }$$

where $\rho_{\lambda }$ represents the hyperspectral reflectance, and ${RSR}_{\lambda }$ denotes the spectral response function (SRF) of a specific sensor. The simulated band-integrated reflectance $\bar{\rho_{\lambda }}$ is calculated by convolving the hyperspectral data with the SRF over the band’s spectral range.

Although the study areas are frequently observed by GF-1/6 and Sentinel-2 satellites, cloud cover masks some pixels and time steps, which requires cloud removal. The *HOT* (Haze Optimized Transformation) index [67,68], calculated as shown in Equation (3), was employed for cloud detection:

(3) HOT=B1−0.5⋅B3

In the *HOT* index, *B1* and *B3* are the blue and red bands. Pixels masked by cloud show higher *HOT* values and can be removed using a thresholding method. GF-1/6 WFV SR data were masked using *HOT*-based cloud detection.

(2) Sentinel-2 MSI

Sentinel-2 Level-2A surface reflectance data on GEE were preprocessed via Sen2Cor. Bands B2, B3, B4, and B8 were scaled (×0.0001) to 0–1 and combined to create reference images. Spatial and temporal subsets matching GF-1/6 WFV data were extracted. The QA60 band was then used to remove opaque and cirrus clouds from Sentinel-2 MSI data [69,70].

Following the cloud-removal process, cloud-free GF-1/6 WFV and Sentinel-2 MSI datasets were prepared. To harmonize the spatial scales—given the 16 m resolution of WFV and 10 m for MSI—Sentinel-2 MSI data were resampled to 16 m using bilinear interpolation to strike a balance between textural detail and computational efficiency. To ensure spatial consistency, global geometric registration was performed using Sentinel-2 MSI as the reference. The registration workflow for GF-1/6 WFV employed Harris corner detection to extract salient feature points, followed by a normalized cross-correlation (NCC) template matching approach to identify optimal conjugate points. Subsequently, an affine transformation was applied to geometrically warp the GF-1/6 WFV data into alignment with the MSI reference. Despite these measures, inherent sensor limitations may cause residual geometric distortions at swath edges and thus reduce the accuracy of subsequent radiometric normalization; this phenomenon is further analyzed in the Results section.

### 3.2. Proposed Radiometric Normalization Framework

**Figure 2:**
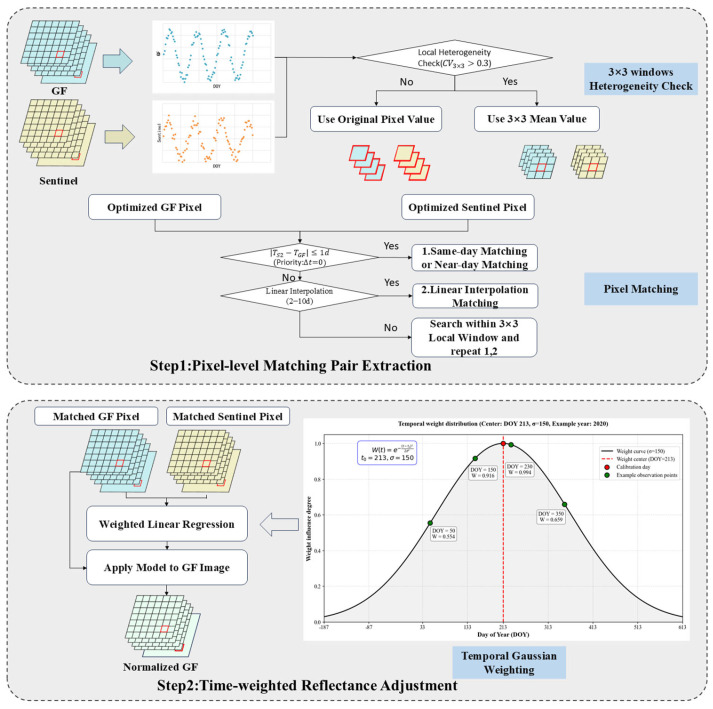
Flowchart of the integrated pixel-level reflectance adjustment method.

The technical workflow of this study is illustrated in Figure 2, which mainly comprises pixel-level matching pair extraction and a time-weighted fitting model. Detailed procedures are presented in the flowchart, with comprehensive descriptions provided in Section 3.2.1 and Section 3.2.2.

#### 3.2.1. Pixel-Level Matching Pair Extraction

To effectively address the geometric distortions challenges inherent in GF-1/6 WFV data, this study introduces the Coefficient of Variation (CV) as a metric for spatial heterogeneity. For the GF time-series imagery, a 3 × 3 moving window was used to calculate the CV of each pixel and provide an initial assessment of local surface heterogeneity.

When the CV exceeded a predefined threshold (CV > 0.3), the pixel was categorized as a highly heterogeneous area. In these cases, the pixel value was reconstructed using the mean reflectance within the window to mitigate the impact of potential geometric offsets. For pixels in homogeneous areas, their original reflectance values were preserved.

The threshold of 0.3 was determined through sensitivity analysis and empirical optimization, and results indicated that this value achieved optimal geometric consistency. Furthermore, the 3 × 3 window was selected based on detail preservation and computational efficiency. Experimental results demonstrated that this size was sufficient to capture the required matching information while maintaining high computational efficiency.

After performing CV heterogeneity detection on each pixel in the image, we implemented a pixel matching strategy for the time series pixels. The detailed principles for the pixel matching strategy are as follows: Data for each pixel were extracted from the time-series images within the study areas. Let $G_{t}\left(i,j\right)$ and $S_{t}\left(i,j\right)$ represent the time-series reflectance of a GF-1/6 pixel and its corresponding Sentinel-2 reference pixel at a specific location $\;\left(i,j\right)$. For pixel-level matching, Sentinel-2 images acquired on the same day as the GF-1/6 data were prioritized ${(G}_{t},S_{t})$. If same-day observations were unavailable, images within a ±1-day window $\;\left(S_{t\pm 1}\right)$ were used to establish the match. In cases where the number of valid temporal pixel pairs was fewer than four, an interpolation strategy was implemented. The target Sentinel-2 value $\left({\hat{S}}_{t}\right)$ was estimated using observations acquired within a 2–10 day window before and after the GF-1/6 acquisition date. This 10-day threshold aligned with the revisit cycle of the Sentinel-2 MSI sensor, which ensured the validity of the spectral information [44]. During this process, only the two closest scenes in time within the 2–10 day range were selected for interpolation. Furthermore, if a pixel still lacked sufficient matching pairs, it was supplemented by its 3 × 3 neighborhood using Methods 1 and 2 described in Step 1 of Figure 2. This hierarchical approach ensured the generation of robust, pixel-level matches across the study area.

Pixel-level reflectance adjustment exhibits high sensitivity to residual geometric misregistration. Despite the registration of GF-1/6 and Sentinel-2 images, residual geometric distortions inevitably remain due to topographic relief and differences in sensor observation geometry. In homogeneous areas, the spectral consistency between sensors is highly robust to minor geometric offsets. In areas with high spatial heterogeneity, even slight geometric offsets can lead to severe radiometric discrepancies between paired pixels. Therefore, this study introduced a spatial heterogeneity detection mechanism to improve geometric robustness in the face of geometric distortions in GF-1/6 WFV.

#### 3.2.2. Time-Weighted Fitting Model

To ensure the effectiveness of the reflectance adjustment, this study implemented a time-decay weighting strategy centered on the sensor calibration date. By using a Gaussian kernel function [71],

(4) $$W=e^{-\frac{\Delta d^{2}}{2\sigma^{2}}}$$

the model assigns higher weights to observations closer to the calibration date, where Δd denotes the temporal offset (time interval from the calibration date), the parameter σ represents the bandwidth of the Gaussian kernel, which controls the decay rate of the weight with temporal offset Δd. In this study, for Areas 1–4 (GF-1 WFV, 2020), the weight center was set to the officially released calibration date of 25 July 2020; for Areas 5–7 (GF-6 WFV, 2022), it was set to 1 August 2022. The temporal weighting parameter σ was determined through experiments conducted within the range of 30 to 360, with σ = 150, yielding the optimal performance across all metrics. At σ = 150, the Gaussian weight decays to 0.5 at approximately ±180 days, which defines an effective half-weight temporal scale covering one year. This aligns with the annual update cycle of the calibration coefficients for the GF satellites. As the IPRA method was implemented within each annual calibration cycle, this parameter setting is physically reasonable.

Based on the above temporal weighting strategy, a weighted linear regression model was employed to characterize the relationship between the extracted time-series pixel pairs $\left(G_{i},S_{i}\right)\to \left(x_{i},y_{i}\right),i=1,\;2,\;3,\;4\ldots \;N$. To account for the radiometric instability and temporal decay of the GF-1/6 WFV sensors, we assumed that observations nearest to the calibration date exhibited the highest reliability. Accordingly, the weight at the calibration date was set to 1, and weights for other dates decayed symmetrically as the temporal offset increased. For each pixel’s time series, the regression coefficients were derived by minimizing a weighted cost function [72],

(5) $$J\left(a,b\right)=\frac{1}{N}\sum_{i=1}^{N}\omega_{i}{\left(y_{i}-{ax}_{i}-b\right)}^{2}$$

where $\omega_{i}$ denotes the weight for each observation at time i; $x_{i}$, $y_{i}$ represent the GF and Sentinel reflectance values at time i, respectively. This approach effectively incorporated temporal decay characteristics and ensured that the fitting process was robust to temporal decay.

### 3.3. Criteria for Performance Assessment

This study comprehensively validated the performance of the proposed integrated pixel-level reflectance adjustment method in terms of accuracy, geometric robustness, and radiometric stability. Furthermore, to evaluate radiometric accuracy, we compared our method with three radiometric normalization methods: IRMAD, HM and TRA.

This study adopted the following metrics to evaluate the performance of the proposed radiometric normalization method: Normalized Absolute Error (NAE), Structural Content (SC), Peak Signal-to-Noise Ratio (PSNR), and Root Mean Square Error (RMSE). Additionally, correlation analysis based on scatter plots was performed for visual consistency verification. The corresponding formulations are presented in Equations (6)–(9) [73].

(6) $$NAE=\frac{\sum_{j=1}^{N}\left|R_{j}-S_{j}\right|}{\sum_{j=1}^{N}\left|R_{j}\right|}$$

(7) $$SC=\frac{\sum_{j=1}^{N}{\left(R_{j}\right)}^{2}}{\sum_{j=1}^{N}{\left(S_{j}\right)}^{2}}$$

(8) $$PSNR=10{log}_{10}\left(\frac{{\left(2^{n_{b}}-1\right)}^{2}}{\frac{1}{N}\sum_{j=1}^{N}{\left(R_{j}-S_{j}\right)}^{2}}\right)$$

(9) $$RMSE=\sqrt{\frac{1}{N}\sum_{j=1}^{N}{\left(R_{j}-S_{j}\right)}^{2}}$$

Here, N denotes the total number of pixels, $R_{j}$ represents the pixel value of the reference image (Sentinel-2 in this study), $S_{j}$ denotes the pixel value of the image to be normalized (GF-1/6), and $n_{b}$ indicates the number of bits per pixel. The Normalized Absolute Error (NAE) measures the relative absolute error between the corrected image S and the reference image R, values closer to zero indicate higher consistency with the reference. Structural Content (SC) quantifies the overall structural similarity between the corrected and reference images and reflects whether the global structure and grayscale energy are preserved. The Peak Signal-to-Noise Ratio (PSNR) assesses the ratio of the signal (i.e., true image) to noise (error) in decibels (dB). Root Mean Square Error (RMSE) represents the square root of the mean squared error, which directly measures pixel-level deviations and provides a direct quantitative measure of normalization performance. Therefore, better radiometric normalization performance is indicated by lower NAE, SC values closer to 1, higher PSNR, and lower RMSE.

In addition to accuracy metrics, we evaluated the performance of the IPRA method in mitigating the inherent spatial misregistration of GF-1/6 data. This evaluation focused on the ability of the IPRA method to maintain radiometric consistency and geometric robustness. Furthermore, to verify the long-term reliability of the method, its radiometric stability was rigorously tested using multi-temporal image sequences. This assessment shifted the focus from single-image-pair validation to long-term consistency and verified the ability of the IPRA method to compensate for sensor-related anomalies such as radiometric decay caused by on-orbit aging. Through the analysis of these sequences, we demonstrate that the proposed method can effectively maintain long-term radiometric consistency.

## 4. Results

### 4.1. Radiometric Differences Between GF-1/6 WFV and Sentinel-2 Imagery

**Figure 3:**
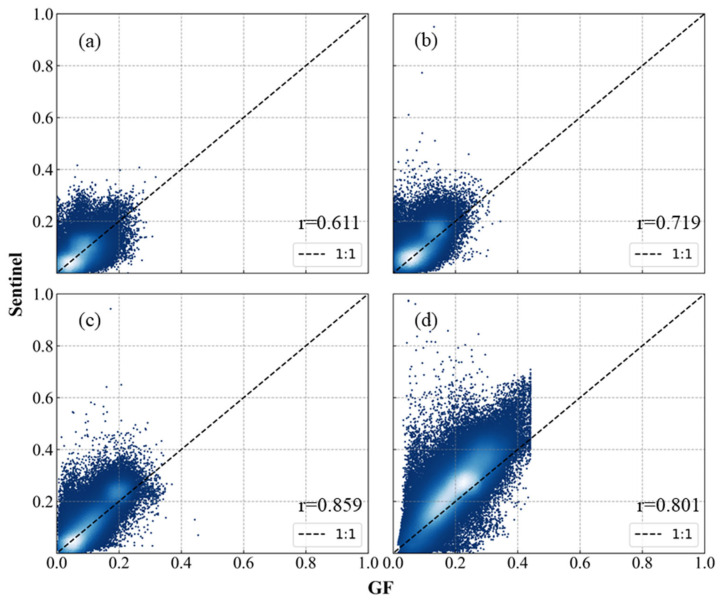
Scatter plot of GF-1/6 WFV and Sentinel-2 MSI pixels across study areas. (**a**), (**b**), (**c**), and (**d**) represent the Blue, Green, Red, and NIR bands, respectively.

According to the scatter plots of different bands across the study areas (Figure 3), the surface reflectance of visible bands ranges from 0 to 0.4, and that of near-infrared bands ranges from 0 to 0.6, covering typical land cover types. Although the overall distributions of GF-1/6 WFV and Sentinel-2 MSI are similar, significant discrepancies exist among different bands: the correlation increases from the blue band to the red band, and the consistency of the near-infrared band is between that of the visible bands. Additionally, GF data exhibit obvious underestimation in the high-value range of 0.4–0.6.

**Figure 4:**
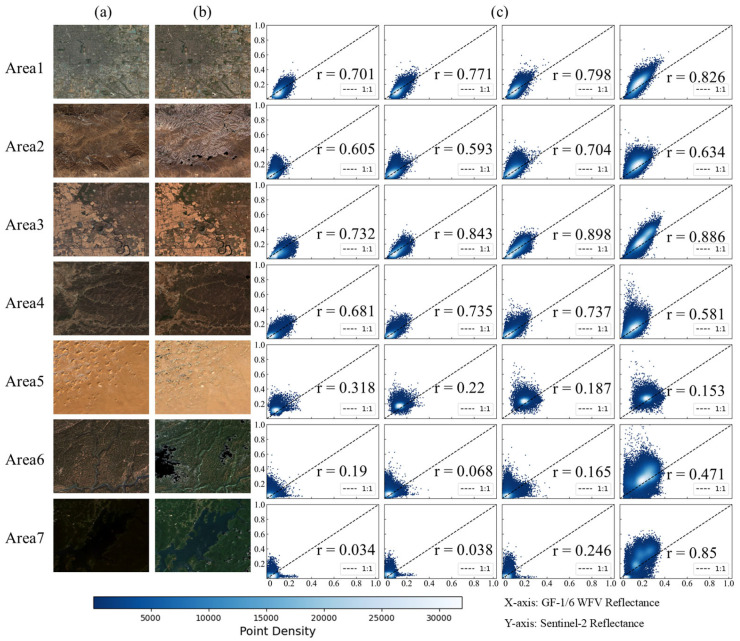
True color composite and scatter plots of each band for GF-1/6 WFV and Sentinel-2 MSI across study areas. Panels (**a**) and (**b**) show the GF-1/6 WFV and Sentinel-2 MSI images, respectively; panel (**c**) illustrates scatter plots of the visible and near-infrared bands, with the *x*-axis representing GF-1/6 WFV and the *y*-axis representing Sentinel-2 MSI. In (**c**), each column corresponds to the Blue, Green, Red, and NIR bands, respectively, and the dashed line represents the 1:1 line.

Based on the analysis in Figure 4 and Table 3, the radiometric consistency across areas exhibits significant spatial heterogeneity. This phenomenon is supported by the quantitative metrics: most areas have NAE > 0.25, RMSE > 0.04, with SC deviating from the ideal value of 1. Furthermore, the average PSNR is approximately 25 dB, which further indicates insufficient radiometric consistency and fidelity in the original images. The above qualitative and quantitative analyses collectively demonstrate significant radiometric inconsistency between the original GF-1/6 WFV and Sentinel-2 MSI, indicating the necessity of subsequent radiometric normalization for GF-1/6 WFV.

### 4.2. Accuracy and Visual Consistency

**Figure 5:**
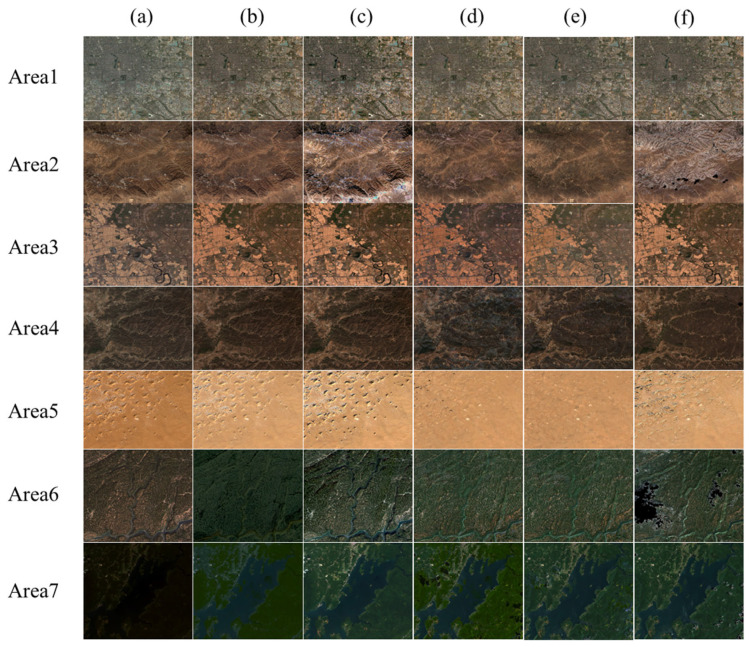
Corrected images by different methods (stretch range: 0–0.3): columns represent (**a**) original GF, (**b**) IRMAD, (**c**) HM, (**d**) TRA, (**e**) IPRA, and (**f**) Sentinel; rows correspond to 7 study areas.

To evaluate the radiometric performance of the IPRA correction, three normalization methods—IRMAD, HM and TRA—were selected as benchmarks for comprehensive comparison. Qualitative results in Figure 5 show that all methods achieve effective radiometric normalization and reduce tonal and visual inconsistencies between the images.

**Figure 6:**
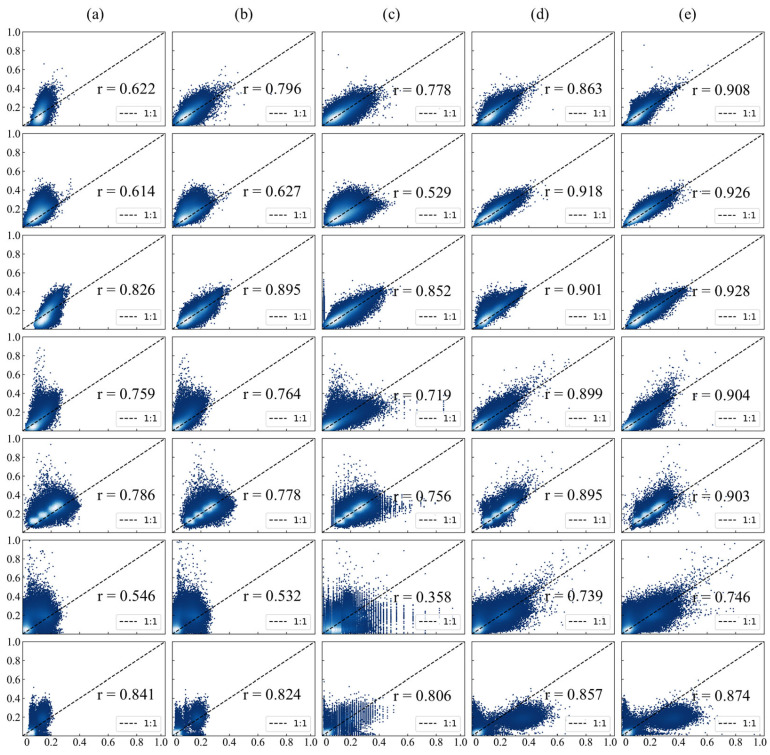
Scatter plots of GF-1/6 WFV images against Sentinel-2 MSI: columns represent (**a**) original GF, (**b**) IRMAD, (**c**) HM, (**d**) TRA, and (**e**) IPRA; rows correspond to 7 study areas.

Furthermore, quantitative evaluations confirm the superior performance of the proposed IPRA method. As shown in Figure 6 and Table 4, the correlation between GF-1/6 WFV and Sentinel imagery was greatly improved after IPRA correction; the scatter points cluster more tightly and align more closely with the 1:1 reference line. Compared to the baseline methods, IPRA achieved the most favorable results across all metrics: NAE decreased significantly from 0.319 to 0.182, representing a reduction of 42.9%, while RMSE decreased from 0.051 to 0.032, a reduction of 37.3%. These results indicate a substantial reduction in global radiometric bias. In terms of structural fidelity, the SC value improved from 1.540 to 1.001, reducing deviation from the ideal value of 1.0 to only 0.001. This indicates a high level of structural consistency between the two sensors. Furthermore, the PSNR increased from 25.906 dB to 30.195 dB. This significant improvement verifies that the IPRA method effectively improves the data quality of GF-1/6 WFV and its consistency with Sentinel-2 MSI.

**Figure 7:**
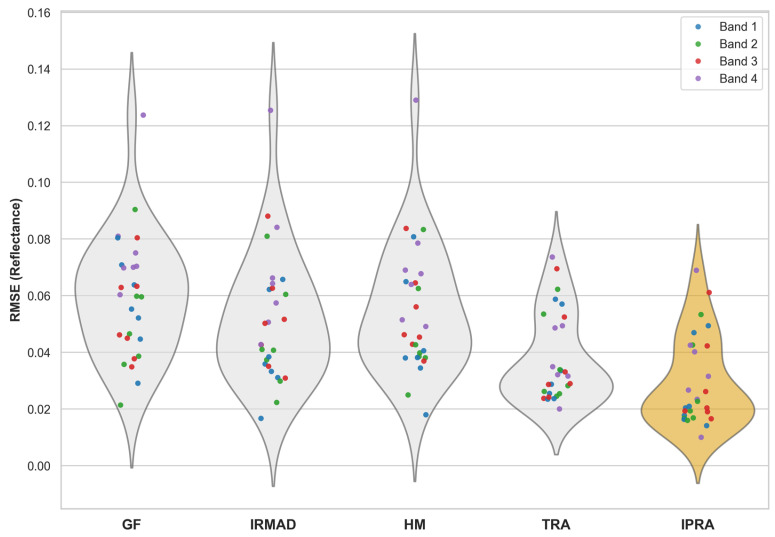
Comparison of metric distributions across different methods and bands (violin–scatter plots).

In addition, we selected GF-1/6 WFV and Sentinel-2 MSI image pairs at representative dates to evaluate the RMSE distributions across different areas, bands, and correction methods. The Kruskal–Wallis test and Dunn’s post hoc comparisons revealed significant differences among the methods across all spectral bands (*p* < 0.001; see Figure 7). Notably, the proposed IPRA method achieved the lowest overall RMSE, demonstrating its superior ability to reduce radiometric discrepancies across all spectral bands. These findings statistically validate the significant advantages of the IPRA method for GF-1/6 WFV radiometric normalization.

**Figure 8:**
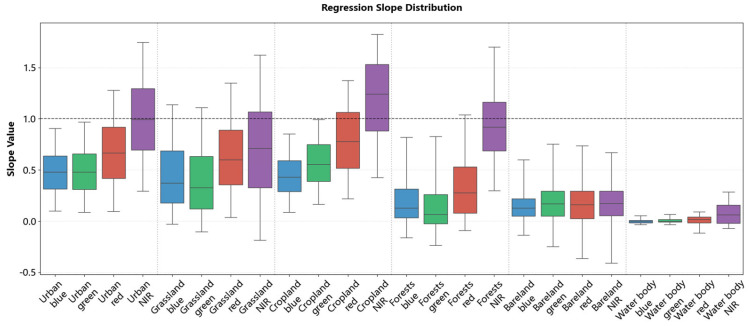
Statistical distribution of regression slopes across different land cover types.

**Figure 9:**
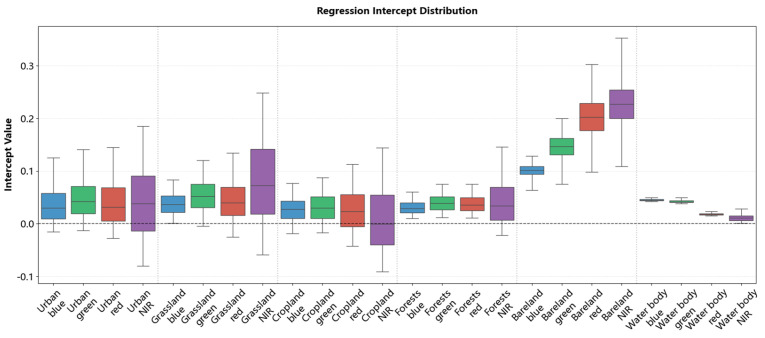
Statistical distribution of regression intercepts across different land cover types.

**Figure A1:**
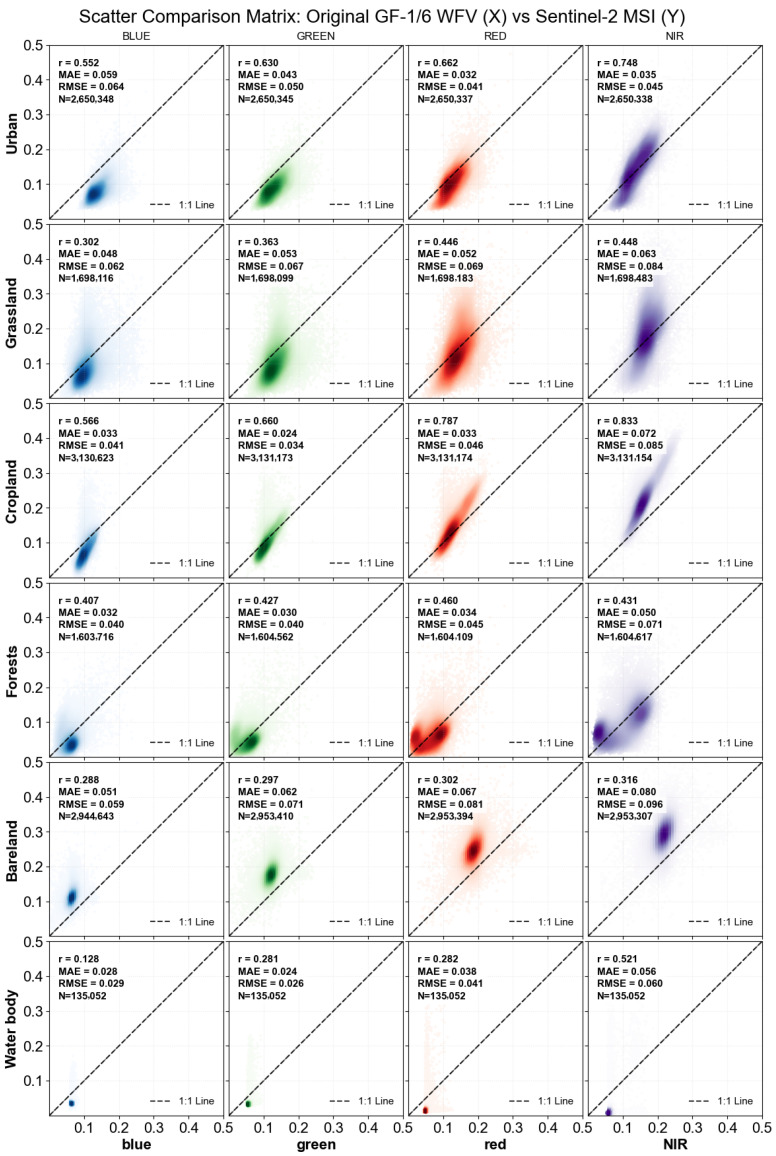
Scatter plots of original GF-1/6 WFV versus Sentinel-2 MSI reflectance across different land cover types.

**Figure A2:**
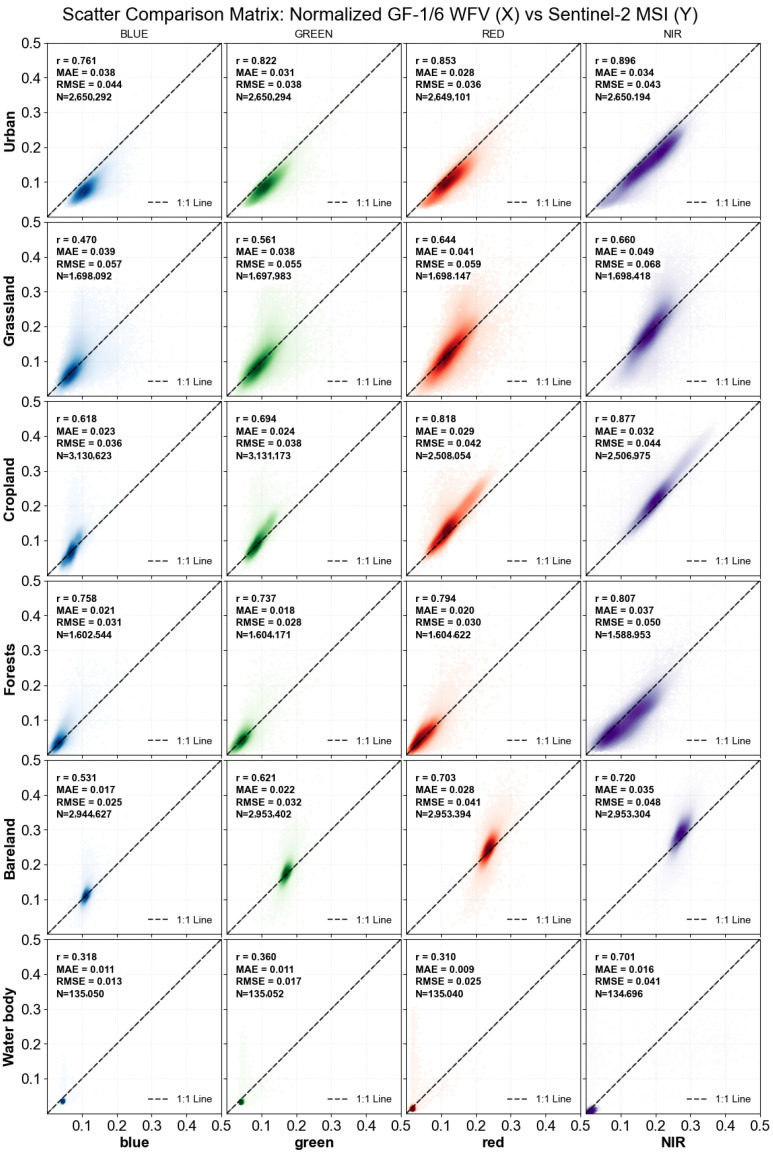
Scatter plots of IPRA-normalized GF-1/6 WFV versus Sentinel-2 MSI reflectance across different land cover types.

To illustrate the pixel-level normalization approach used in this study, we present the spatial distributions of regression coefficients (slopes in Figure 8 and intercepts in Figure 9) across typical land cover types, including urban areas, grassland, cropland, forests, bareland, and water bodies. Supplementary scatter plots in Appendix A, Figure A1 and Figure A2 show the correlation between GF-1/6 WFV and Sentinel-2 MSI before and after normalization.

The scatter plot results revealed that for urban areas, grassland, cropland, forests, and water body, the original GF-1/6 WFV reflectance values were generally higher than those of Sentinel-2 MSI. Notably, some pixels showed a reversal in the NIR band, where GF values were lower than Sentinel-2 values. This is consistent with the slope distributions: these areas showed slopes < 1 and intercepts near 0 in visible bands (R, G, B), and slopes > 1 in the NIR band.

In contrast, bareland displayed a distinct radiometric pattern. Although the raw GF-1/6 WFV reflectance was consistently lower than that of Sentinel-2 across all bands, the regression was characterized by small slopes (0–0.25) and large intercepts (>0.1, and >0.2 in the red and NIR bands). This indicates that the reflectance adjustment for bareland was dominated by intercept offsets rather than linear scaling. These findings indicate that regression coefficients are highly sensitive to land cover type; even within the same class, the coefficients show obvious cross-band and spatial variability.

### 4.3. Geometric Robustness

Geometric distortions are a common issue that can induce pixel mismatching and thus reduce the accuracy of radiometric normalization. GF-1/6 WFV data exhibit obvious geometric distortions, especially in Areas 2, 3, 4, and 6, where the geometric RMSE values exceed 1 pixel.

To evaluate the geometric robustness of different normalization methods, we performed a geometric offset analysis across the study areas. By examining the spatial alignment of key land cover edges and structures in GF-1/6 WFV images before and after correction and Sentinel-2 reference images, we analyzed the geometric offsets before and after correction and quantified them as RMSE. The AKAZE feature detector [74] was used to extract matching point pairs between the uncorrected, corrected images and the Sentinel-2 images. The geometric RMSE was then calculated from these positional offsets. Table 5 presents the geometric RMSE values for different areas before and after correction.

The results indicate that the IPRA method substantially improves the geometric alignment between GF-1/6 WFV and the reference imagery. Quantitatively, the average geometric offset across all study areas decreased from 1.093 pixels to 0.520 pixels, representing an overall improvement of approximately 52.4%. Notably, all post-correction offsets were reduced to below 1.0 pixels, which demonstrates that the proposed method consistently achieves robust sub-pixel geometric alignment.

**Figure 10:**
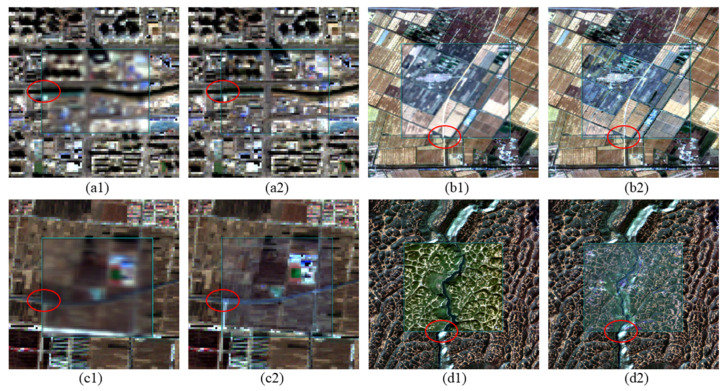
Geometric deviation performance after radiometric normalization in different study areas (Area 1, 3, 4, and 6). The backgrounds are Sentinel-2 reference images; the centers of (**a1**–**d1**) represent the original GF-1/6 WFV images showing initial deviations, while the centers of (**a2**–**d2**) represent the images processed using the IPRA method. The red circles mark the locations of obvious geometric deviations.

The correction performance is particularly notable in areas with complex textures. For instance, geometric offsets in cropland (Area 3), grassland (Areas 2 and 6) were reduced by 1.093, 0.948, and 0.831 pixels, respectively. Such areas often present challenges for traditional radiometric normalization methods due to geometric errors. By using spatial information within a 3 × 3 local window, the proposed IPRA method shows strong tolerance to geometric errors. The adjusted results demonstrate strong geometric consistency with Sentinel-2 MSI, see Figure 10.

### 4.4. Radiometric and Temporal Stability

**Figure 11:**
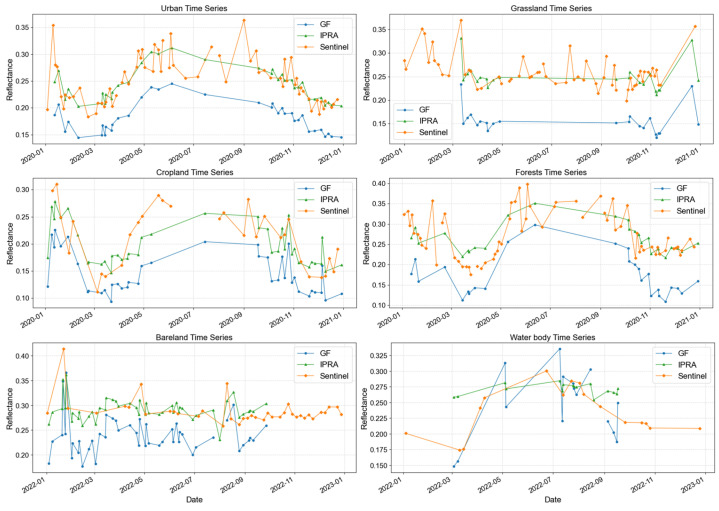
NIR band time-series reflectance results of original GF-1/6 WFV, IPRA-normalized imagery, and Sentinel-2 MSI across various land-cover types.

**Figure A3:**
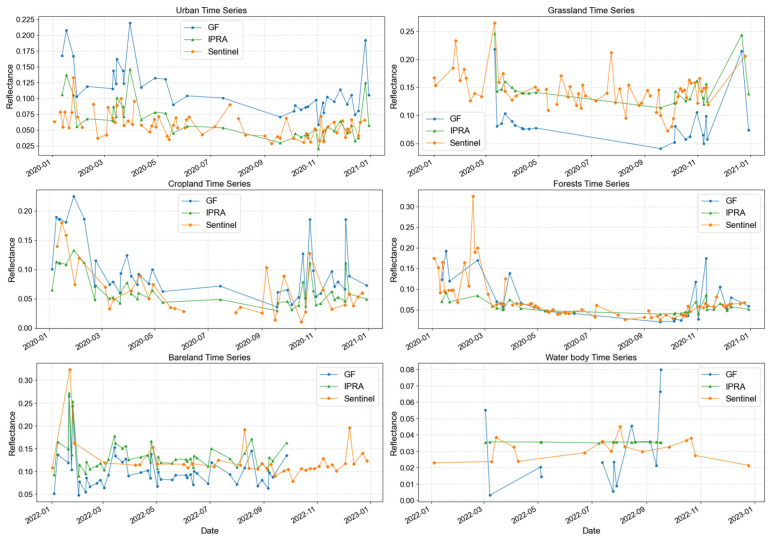
Blue band time-series reflectance results of original GF-1/6 WFV, IPRA-normalized imagery, and Sentinel-2 MSI across various land-cover types.

**Figure A4:**
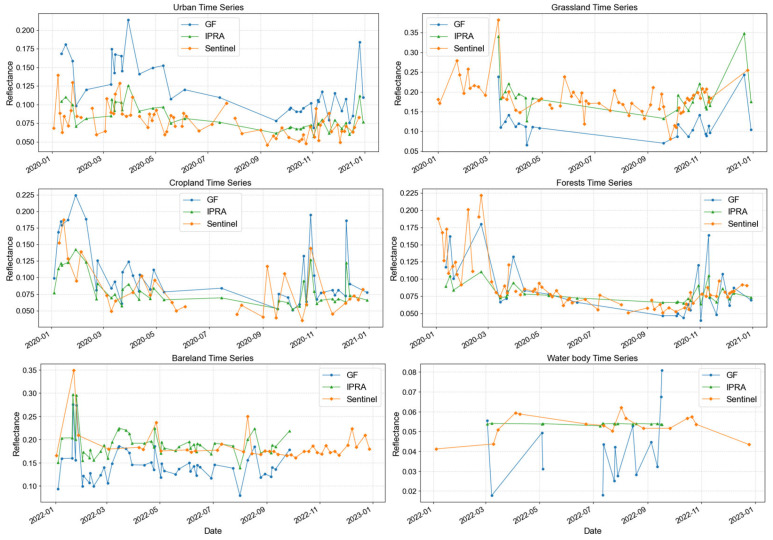
Green band time-series reflectance results of original GF-1/6 WFV, IPRA-normalized imagery, and Sentinel-2 MSI across various land-cover types.

**Figure A5:**
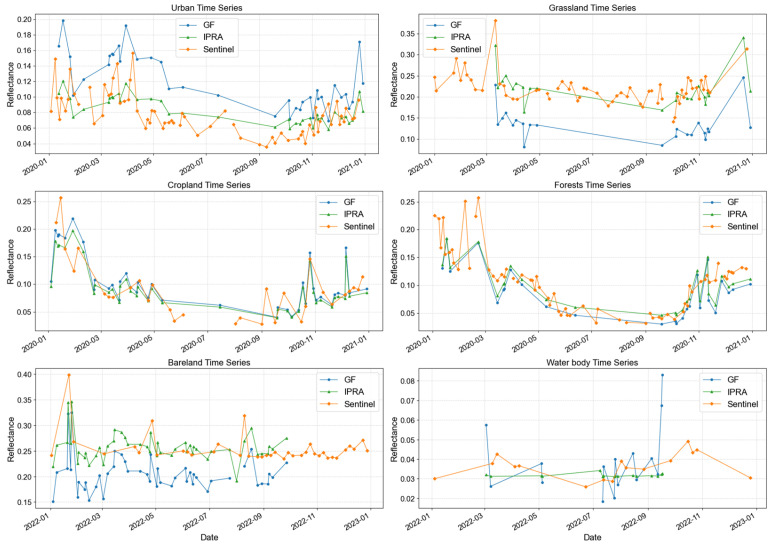
Red band time-series reflectance results of original GF-1/6 WFV, IPRA-normalized imagery, and Sentinel-2 MSI across various land-cover types.

To evaluate the long-term stability of the method, we analyzed the reflectance trends of pixels from different land cover types before and after normalization. Figure 11 presents the near-infrared (NIR) band as an example, with other bands provided in Appendix A Figure A3, Figure A4 and Figure A5. The time-series reflectance analysis demonstrates that the IPRA method significantly improves radiometric accuracy and temporal stability across all typical land cover types. As shown in the figures, the raw GF-1/6 WFV data (blue) exhibit systematic deviations (both underestimation and overestimation), whereas the IPRA-corrected curves (green) agree closely with the Sentinel-2 MSI reference (orange) in both magnitude and temporal trend. For land cover types with clear seasonal variation (e.g., cropland and forests), the normalization ensures that the GF-1/6 WFV time-series profiles are consistent with the reference and capture the main phenological stages. Significant radiometric discrepancies were observed between the original GF and Sentinel-2 profiles in urban, grassland, bareland, and water body areas. After IPRA correction, the radiometric quality in these areas was significantly improved, effectively reducing inter-sensor discrepancies. Similarly, in areas with high inherent consistency (e.g., cropland and forests), IPRA maintained high radiometric quality, preserving the original spectral fidelity while ensuring temporal consistency.

## 5. Discussion

### 5.1. Superior Performance of the Proposed IPRA Framework

A comparative analysis reveals that existing methods, including IRMAD, HM, and TRA, face inherent limitations when applied to GF-1/6 WFV imagery. Although the scatter plots of the IRMAD and HM methods align with the 1:1 line, their distributions remain widely scattered; furthermore, the geometric RMSE for these methods remains unsatisfactory. Although the pixel-level adjustment of TRA produces more concentrated scatter plots, it is still affected by geometric offsets. Overall, these methods perform poorly in mitigating the inherent geometric distortions of GF-1/6 WFV data. IRMAD relies on Canonical Correlation Analysis (CCA) and requires precise geometric co-registration. When applied to GF-1/6 WFV imagery characterized by geometric distortions, IRMAD suffers from the misidentification or omission of PIFs. This leads to pseudo-changes induced by geometric offsets rather than real surface variations, thereby producing poor geometric consistency and unreliable normalization. In spatially heterogeneous areas, HM is ineffective because geometric distortions lead to spatial misalignment between corresponding patches. This results in inaccurate source and reference histograms, because the statistical samples no longer represent corresponding areas. Consequently, such histogram matching yields incorrect reflectance adjustments, thus reducing the accuracy of radiometric normalization. TRA relies on simple linear fitting of pixel-level time-series pairs. When applied to GF-1/6 WFV data with geometric distortions, TRA operates on spatially mismatched time-series data. Although it may achieve a mathematical adjustment of the pixel values, it fails to account for geometric errors, which leads to unsatisfactory spatial integrity and low geometric accuracy. In contrast, the proposed IPRA framework incorporates a spatial heterogeneity detection mechanism that uses corresponding pixel information. This method effectively mitigates these geometric distortions and achieves superior radiometric accuracy while ensuring improved geometric consistency.

In addition to the excellent performance mentioned above, the superiority of the proposed IPRA method can also be demonstrated from the perspective of the difference in regression coefficients. The results presented in Section 4.2 indicate that different land cover types exhibit significantly different regression coefficients. Moreover, large variations in coefficients exist even within the same land cover type: the slope (a) varies by more than 2 and the intercept (b) by more than 0.2. This indicates that even for the same land cover class, radiometric discrepancies can vary greatly with location and surface conditions. Existing radiometric normalization methods fail to account for these pixel-specific differences. The IPRA framework addresses these variations and achieves superior radiometric normalization performance. This validates the necessity of the proposed method for accurate cross-sensor radiometric normalization.

### 5.2. Sensitivity Analysis of the Spatial Heterogeneity Detection Mechanism

To mitigate the geometric distortions inherent in GF-1/6 WFV imagery, we implemented a spatial heterogeneity detection mechanism to classify areas according to their heterogeneity levels. We performed a comprehensive sensitivity analysis on Area 1 to evaluate the effects of different window sizes and Coefficient of Variation (CV) thresholds on geometric accuracy.

**Figure 12:**
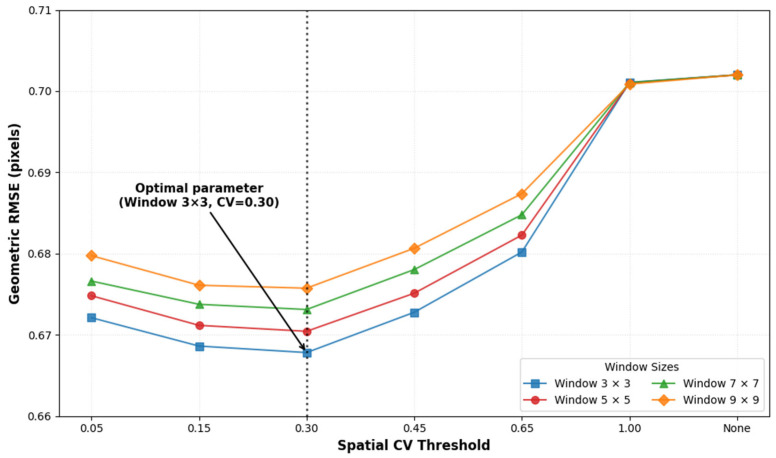
Sensitivity analysis of geometric RMSE across varying spatial CV thresholds and window sizes. The arrow highlights the optimal parameter combination (Window 3 × 3, CV = 0.30).

The results demonstrate that a 3 × 3 window consistently yields the lowest geometric RMSE and achieves superior geometric consistency across the study area. Furthermore, the evaluation of different CV thresholds indicates that CV = 0.3 is the optimal value, corresponding to the minimum RMSE (Figure 12). Notably, the geometric RMSE increased significantly when CV approached 1.0 and when no threshold was applied (which is equivalent to the conventional TRA method). These findings confirm that the proposed IPRA method effectively mitigates geometric distortions specific to GF satellites and outperforms conventional methods in complex areas.

### 5.3. Sensitivity Analysis of the Time-Weighted Fitting Model

Considering the temporal decay of sensor performance over different years, this study proposes a time-weighted fitting model. The core of this model is to correlate the image acquisition date with the official calibration release date of that year, and to set the calibration date as the time-weighted center peak. The weight follows a Gaussian decay function: the smaller the temporal distance between the image and the annual calibration reference point, the higher its weight in the regression model.

**Figure 13:**
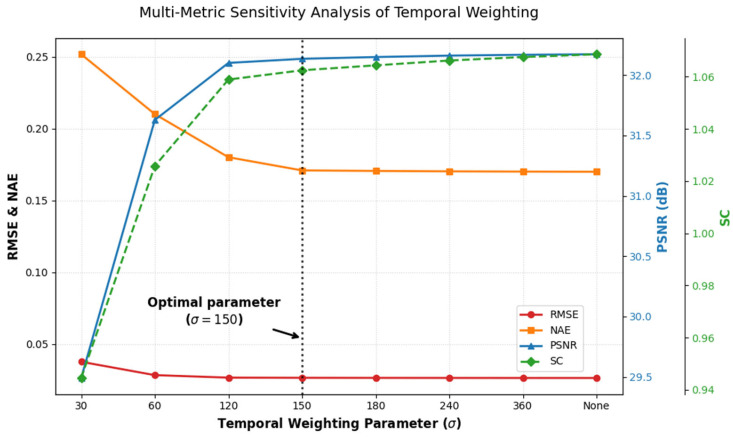
Sensitivity of RMSE, NAE, PSNR, SC to the temporal weighting parameters (σ). The dashed vertical line indicates the optimal parameter selection (σ = 150).

To determine the optimal value of the temporal weighting parameter σ, a sensitivity experiment was conducted. Performance was evaluated in Area 1 using four metrics: NAE, RMSE, SC, and PSNR (Figure 13). The results indicate that when σ < 100, the model is very sensitive to short-term fluctuations near the calibration date, which leads to a significant increase in error. In contrast, when σ > 200, the Gaussian weighting function flattens, greatly increasing the weight of distant observations. This causes the regression to approximate ordinary least squares (OLS). As a result, both the SC index and histogram consistency gradually degrade, which demonstrates ineffective compensation for sensor temporal decay. As σ approaches 150, NAE, RMSE, and PSNR exhibit a clear saturation trend and reach relative stability. The SC index rises further beyond this threshold and demonstrates a gradual deviation from the reference. Thus, σ = 150 is determined as the optimal parameter value.

**Figure 14:**
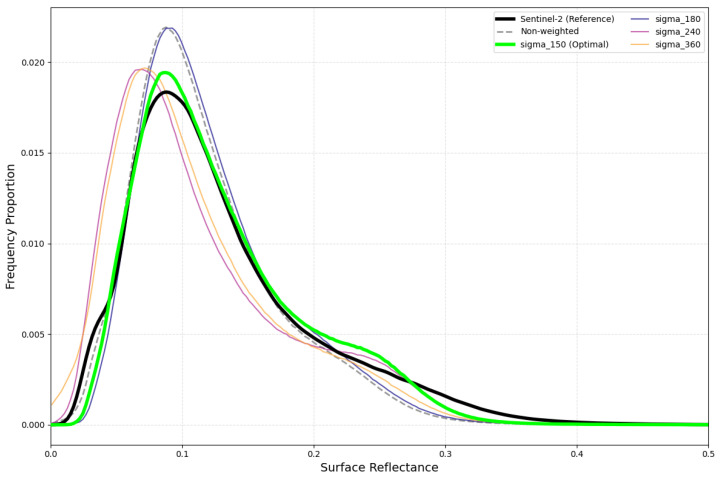
Sensitivity of surface reflectance frequency distributions to varying temporal weighting parameter (σ) in the IPRA method. The green curve (σ = 150) exhibits the highest degree of similarity to the Sentinel-2 MSI reference distribution, identifying it as the optimal parameter selection for the IPRA method.

To investigate this, we analyzed the frequency distribution curves for σ = 150 and higher values (Figure 14). The plots show that when σ > 150, the proportion of low-reflectance pixels increases, as shown by the leftward shift in the frequency curves. This shift is directly related to the continued rise in the SC index, which suggests that there may be an anomalous adjustment at a higher σ level. Therefore, we determined that σ = 150 serves as the optimal parameter. It balances error stability and structural consistency, and delivers the most robust radiometric normalization performance.

### 5.4. Performance of IPRA in Temporal Radiometric Dynamics

Time series analysis shows that the proposed IPRA method effectively reduces the long-term radiometric differences between GF-1/6 WFV and Sentinel-2 MSI while maintaining natural seasonal variations. As illustrated in Figure 11, GF-1/6 WFV data capture the seasonal variations in surface reflectance, which correspond to the inherent phenological changes in land covers in urban areas, cropland, and forests. After IPRA correction, the reflectance curves not only align more closely with Sentinel-2 MSI but also preserve these real seasonal phenological characteristics. The proposed method mainly corrects long-term radiometric drift induced by sensor effects, while preserving genuine seasonal variations. In this way, it ensures robust long-term radiometric consistency.

However, analysis across urban areas, grassland, and forests reveals that the land surface may exhibit abrupt and rapid changes within regular phenological cycles. These fluctuations are difficult to predict. Existing temporal interpolation methods thus fail to capture such abrupt changes, and their results may deviate from true values and reduce normalization accuracy. Therefore, improving the ability to capture rapid surface changes is a key priority for future work, which can be addressed by incorporating satellite data with higher temporal resolution.

## 6. Conclusions

To address the challenges caused by geometric distortions and temporal decay in GF-1/6 WFV sensors, this study proposes an integrated pixel-level reflectance adjustment method combining spatial heterogeneity detection mechanism with a time-weighted fitting model. The proposed IPRA method employs two strategies to address the problems of geometric distortions and temporal decay, respectively.

As for the geometric distortions, the method employs a spatial heterogeneity detection mechanism and performs neighborhood averaging for high-variance areas. This mechanism improves tolerance to geometric distortions and maintains the geometric fidelity of the imagery. As for the temporal decay, a Gaussian weighting model centered on official calibration dates is developed. It effectively mitigates the issues caused by sensor decay over time. By combining these two strategies, the proposed IPRA framework enables reliable radiometric normalization under the given constraints.

Quantitative comparisons with conventional methods further validate the advantages of the proposed IPRA framework. Compared with conventional methods (i.e., IRMAD, HM, and TRA), the integrated design of IPRA simultaneously reduces geometric errors and corrects radiometric discrepancies. Quantitative results verify its superior performance across multiple metrics: the NAE decreases from 0.319 to 0.182, the RMSE is reduced from 0.051 to below 0.032, the PSNR exceeds 30 dB, and the SC converges to 1. These results confirm that the proposed method effectively addresses the inherent constraints of GF-1/6 WFV radiometric normalization. IPRA thus offers a robust and reliable solution for quantitative remote sensing applications of Chinese GF-1/6 WFV data.

## Figures and Tables

**Table 1:** Summary of the selected study areas.

Study Areas	Latitude (°N)	Longitude (°E)	Land Cover
Area 1	39.88	116.45	Urban
Area 2	40.29	114.37	Grassland
Area 3	39.52	117.71	Cropland
Area 4	40.50	117.27	Forests
Area 5	40.68	102.48	Bareland
Area 6	30.50	110.14	Grassland
Area 7	25.90	113.41	Water body

**Table 2:** Summary of Satellite Data. GF indicates GF-1/6 WFV; S2 indicates Sentinel-2 MSI; count means annual number of available scenes used for analysis.

Study Areas	Year	GF Count	S2 Count	GF Type
Area 1	2020	37	71	GF1 WFV
Area 2	2020	24	70	GF1 WFV
Area 3	2020	40	32	GF1 WFV
Area 4	2020	26	72	GF1 WFV
Area 5	2022	50	39	GF6 WFV
Area 6	2022	14	19	GF6 WFV
Area 7	2022	17	16	GF6 WFV

**Table 3:** Evaluation metrics before radiometric normalization between original GF-1/6 WFV and Sentinel-2 MSI across all study areas.

Study Areas	NAE	SC	PSNR (dB)	RMSE
Area 1	0.259	1.289	26.915	0.045
Area 2	0.285	1.618	26.389	0.048
Area 3	0.276	1.273	26.402	0.048
Area 4	0.331	1.418	27.607	0.042
Area 5	0.205	1.484	26.033	0.050
Area 6	0.360	1.711	23.507	0.067
Area 7	0.519	1.985	24.487	0.060

**Table 4:** Evaluation metrics of different normalization methods.

	NAE	SC	PSNR (dB)	RMSE
Raw GF	0.319	1.540	25.906	0.051
IRMAD	0.231	1.225	27.971	0.041
HM	0.238	0.993	27.718	0.043
TRA	0.193	1.008	29.974	0.034
IPRA	**0.182**	**1.001**	**30.195**	**0.032**

**Table 5:** Comparison of geometric RMSE (pixels) between the original GF data and various normalization methods (IRMAD, HM, TRA and IPRA) across study areas.

Areas	Original	IRMAD	HM	TRA	IPRA
Area 1	0.881	0.841	0.891	0.703	**0.668**
Area 2	1.312	0.521	0.896	0.624	**0.364**
Area 3	1.89	1.079	1.023	1.124	**0.797**
Area 4	1.349	1.655	1.229	0.973	**0.608**
Area 5	0.848	1.025	0.861	1.641	**0.763**
Area 6	1.188	1.543	1.666	0.373	**0.357**
Area 7	0.182	0.384	1.483	0.346	**0.086**

## Data Availability

The datasets presented in this article are not readily available because the data are part of an ongoing research project and are currently being used for further analysis. Requests to access the datasets should be directed to Jianli Shi (shijianli23@mails.ucas.ac.cn).
